# Fixed-point iterative linear inverse solver with extended precision

**DOI:** 10.1038/s41598-023-32338-5

**Published:** 2023-03-30

**Authors:** Zheyuan Zhu, Andrew B. Klein, Guifang Li, Sean Pang

**Affiliations:** CREOL, College of Optics and Photonics, University of Central Florida, Orlando, FL 32816 USA

**Keywords:** Electrical and electronic engineering, Computational science, Applied mathematics

## Abstract

Solving linear systems, often accomplished by iterative algorithms, is a ubiquitous task in science and engineering. To accommodate the dynamic range and precision requirements, these iterative solvers are carried out on floating-point processing units, which are not efficient in handling large-scale matrix multiplications and inversions. Low-precision, fixed-point digital or analog processors consume only a fraction of the energy per operation than their floating-point counterparts, yet their current usages exclude iterative solvers due to the cumulative computational errors arising from fixed-point arithmetic. In this work, we show that for a simple iterative algorithm, such as Richardson iteration, using a fixed-point processor can provide the same convergence rate and achieve solutions beyond its native precision when combined with residual iteration. These results indicate that power-efficient computing platforms consisting of analog computing devices can be used to solve a broad range of problems without compromising the speed or precision.

## Introduction

Floating-point digital processing is the standard computing paradigm in virtually all scientific and engineering applications, such as image processing^1–3^, compressive sensing^4–6^, and predictive control systems^7–9^. The cores of these applications often involve large-scale linear systems, which require multiplying and/or inverting matrices with thousands to millions of elements. A conventional digital floating-point processor, such as a single CPU core, processes the matrix multiplication as sequential multiply-accumulate (MAC) operations, which are not efficient in handling large-scale linear systems and could yield minutes to hours of computation time even for a moderate-scale problem^10,11^.

Driven by the growing demand for data processing power, digital accelerators that implement a large array of parallel, half-precision floating-point processing units (FPUs), such as Google TPU^12^ and the Tensor Cores in NVIDIA Volta GPU^13^, have been developed, particularly for artificial intelligence applications. Due to the on-chip power consumption limit, fixed-point digital MAC unit that requires only a fraction of the energy of the FPU^14,15^ becomes increasingly favorable in large-scale digital accelerators. Recently, analog accelerators that utilize natural physical processes for array multiplications and summations have shown to be even faster and more energy-efficient for matrix processing^16^, with implementations on both electronic^17,18^ and photonic^19,20^ platforms. Yet, analog accelerators are susceptible to signal artifacts arising from noises and device imperfections^21^, and are unable to achieve floating-point precision. As a result, fixed-point is the de-facto data format in both analog and digital accelerators^22^.

Currently, the applications of analog or digital fixed-point accelerators are limited to problems robust against computing errors, such as neural network inference^23^, adaptive filtering^24^, and binary optimization^25^. On the other hand, many computing tasks in science and engineering are inverse problems without analytical solutions, which must be solved iteratively. For these problems, floating-point precision is typically required to cache the intermediate results^26,27^, because low-precision matrix calculations can introduce cumulative errors and stall the iterative process. The precision requirement excludes many resource-demanding computing applications from deployment on analog or digital fixed-point accelerators.

Extending the iterative linear solver to fixed-point data format can effectively leverage the high speed and efficiency of analog accelerators for a broad range of computing problems in science and engineering. However, the errors of fixed-point arithmetic and the precision of the result in both the forward and inverse processes of a linear system have not been quantitatively studied. This work examines the properties of an iterative linear inverse algorithm (Richardson iteration) in which the matrix computations are performed with fixed-point arithmetic. Using residual iterations for error correction, the precision of the iterative solution can be theoretically extended to any desired level. Similar methods have been previously proposed for correcting the computing errors in reduced precision floating-point processors^28,29^ or hybrid analog–digital processors^30,31^, but quantitative analyses on the error and convergence of a fixed-point solver are still absent. Here we introduce two theorems describing the convergence criteria and error of a fixed-point Richardson solver. The theorems have two major implications: (1) the fixed-point solver does not compromise the convergence rate, and (2) using residual iterations, the fixed-point solver can reach solutions beyond its native precision limit. We verify the two theorems with three examples, namely, matrix inversion, image deconvolution, and tomography reconstruction. We also present a prototype fixed-point iterative solver implemented on a field programable gate array (FPGA), which reaches the solution in the same number of iterations as a floating-point CPU and reduces the calculation time by two folds.

## Theory

### Iterative solution of linear system

The problem of solving a linear system aims to deduce the inputs, $\mathbf{x}$, that generate the observations, $\mathbf{y}$, given the physical model of the system, expressed by a matrix $\mathbf{A}$. Due to the size of the system matrix and noises in the measurement process, direct inverse of the observations ${\mathbf{y}}$ is usually impractical and, in many cases, impossible. As a result, many inverse algorithms rely on iterative refinements to approximate the solution ^32^. Assuming that the measurements are dominated by Gaussian noise, finding the solution ${\hat{\mathbf{x}}}$ given the observations ${\mathbf{y}}$ amounts to minimizing the objective function ^33^,

1 $$ {\hat{\mathbf{x}}} = \mathop {{\text{argmin}}}\limits_{{\mathbf{x}}} \frac{1}{2}\left| {{\mathbf{y}} - {\mathbf{Ax}}} \right|_{2}^{2} , $$

where $\left| \cdot \right|_{2}$ denotes the *ℓ*^**2**^ norm. The solver starts with an initial guess, ${\mathbf{x}}_{0}$, and moves along the gradient direction, $\partial L/\partial {\mathbf{x}}$, in steps of size $\tau$ to update the estimate. Given the objective function in Eq. (1), the update from step $k$ to $k + 1$ can be expressed as follows,

2 $$ {\mathbf{x}}_{k + 1} = {\mathbf{x}}_{k} - \tau {\mathbf{A}}^{T} \left( {{\mathbf{Ax}}_{k} - {\mathbf{y}}} \right). $$

Equation (2) can be rearranged into the form of Richardson iteration^34^ (Eq. (3)) by introducing a pre-calculated vector ${\mathbf{b}} = \tau {\mathbf{A}}^{T} {\mathbf{y}}$,

3 $$ {\mathbf{x}}_{k + 1} = \left( {{\mathbf{I}} - \tau {\mathbf{A}}^{T} {\mathbf{A}}} \right){\mathbf{x}}_{k} + {\mathbf{b}}, $$

where each iteration comprises two matrix–vector multiplications and two vector additions. In the following sections, we discuss the error and convergence of the iterative linear solver when Eq. (3) is carried out in fixed-point precision available to analog or digital low-precision accelerators.

### Fixed-point format

In the fixed-point data format, a vector, matrix, or tensor is represented by an array of signed mantissas, and a global exponent is shared among all the elements. Each element,$$ x_{i}$$, in the $$L$$-bit fixed-point representation, $${\tilde{\mathbf{x}}}$$, of an $$N$$-element array $${\mathbf{x}} \in {\mathbb{R}}^{N}$$ can be expressed as,

4 $$ \tilde{x}_{i} = {\text{sign}}\left( {x_{i} } \right) \times {\text{mant}}_{i} \times 2^{{{\text{expo}} - \left( {L - 1} \right)}} . $$

Here ${\text{expo}}$ denotes the exponent indicating the number of bits above decimal point. To avoid overflow, the exponent is usually determined by the maximum element of the array,

5 $$  {\text{expo}} = \left\lceil {\log _{2} \mathop {\max }\limits_{i} \left( {{\text{abs}}\left( {x_{i} } \right)~} \right)} \right\rceil , $$

where $x$ denotes the rounding to the nearest integer larger than $x$. The $\left( {L - 1} \right)$-bit mantissas ${\text{mant}}_{i}$ are calculated as,

6 $$  {\text{mant}}_{i}  = \left\lfloor {abs\left( {x_{i} } \right) \times 2^{{L - 1 - {\text{expo}}}} } \right\rfloor ,  $$

where $x$ denotes the rounding to the nearest integer smaller than $x$.

The multiplication between a fixed-point matrix ${\tilde{\mathbf{M}}}$ and a fixed-point vector ${\tilde{\mathbf{x}}}$ can be simplified as integer arithmetic between the mantissas, accompanied by bit-shifting to match the exponent of the result. Compared with the floating-point matrix–vector product ${\mathbf{y}} = {\mathbf{Mx}}$, the *ℓ*^**2**^ norm of the error, ${\updelta }{\mathbf{y}}$, of the fixed-point matrix–vector product ${\tilde{\mathbf{y}}} = {\tilde{\mathbf{M}\tilde{x}}}$ is bounded by,

7 $$ \left| {{\mathbf{\delta y}}} \right|_{2} = \left| {\tilde{\mathbf{M}}{\tilde{\mathbf{x}}} - {\mathbf{Mx}}} \right|_{2} \le \eta \left| {\mathbf{M}} \right|_{2} \left| {\mathbf{x}} \right|_{2} . $$

Here $\left| {\mathbf{M}} \right|_{2}$ denote the *ℓ*^2^ norm of the operator. An upper bound of the coefficient $\eta$ is given by $\zeta_{v} + \zeta_{m} + 3\zeta_{v} \zeta_{m}$ in Section S1 of the supplementary materials. The coefficients $\zeta_{v}$ and $\zeta_{m}$ are defined as:

8 $$ \begin{aligned} & \zeta_{v} : = \left| {{\mathbf{x}} - { }{\tilde{\mathbf{x}}}} \right|_{2} /\left| {\mathbf{x}} \right|_{2} , \\ & \zeta_{m} : = \left| {{\mathbf{M}} - { }{\tilde{\mathbf{M}}}} \right|_{2} /\left| {\mathbf{M}} \right|_{2} , \\ \end{aligned} $$

which represent the normalized rounding errors of the fixed-point vector and matrix, respectively, and are dependent on the bit widths and exponents of the arrays. In most cases, $\eta$ is much lower than this upper bound, depending on the distribution of elements in ${\mathbf{M}}$ and ${\mathbf{x}}$.

### Error and convergence criteria of fixed-point Richardson iteration

The solution ${\mathbf{x}}^{*}$ to a linear system ${\mathbf{y}} = {\mathbf{Ax}}$ is unique if matrix ${\mathbf{A}}$ has full rank. To ensure that the intermediate estimations in the Richardson iteration $\left\{ {{\mathbf{x}}_{k} } \right\}$ converge to the solution ${\mathbf{x}}^{*}$ as the iteration number $k \to \infty$, all the eigenvalues of matrix $\left( {{\mathbf{I}} - \tau {\mathbf{A}}^{T} {\mathbf{A}}} \right)$ must fall within $\left( { - 1,1} \right)$. Since the largest and smallest eigenvalues are $1 - \tau \left| {{\mathbf{A}}^{T} {\mathbf{A}}} \right|_{2} /\kappa$ and $1 - \tau \left| {{\mathbf{A}}^{T} {\mathbf{A}}} \right|_{2}$, respectively, where the operator norm $\left| {{\mathbf{A}}^{T} {\mathbf{A}}} \right|_{2}$ equals the largest eigenvalue, and $\kappa$ is the condition number of ${\mathbf{A}}^{T} {\mathbf{A}}$, the choice of the step size, $\tau$, is confined by:

9 $$ 0 < \tau < \frac{2}{{\left| {{\mathbf{A}}^{T} {\mathbf{A}}} \right|_{2} }}. $$

In practice, to ensure the convergence while expediating the iterative solver, the maximum step size, $\tau_{max}$, is often selected with a safety margin $0 < \chi \ll 1$ as follows:

10 $$ \tau_{max} = \frac{2 - \chi }{{\left| {{\mathbf{A}}^{T} {\mathbf{A}}} \right|_{2} }}. $$

For fixed-point Richardson iterations, Theorem 1 below summarizes the error and convergence criteria based on the fixed-point matrix multiplication error $\eta$.

#### ***Theorem 1:***

For a linear system ${\mathbf{Ax}} = {\mathbf{y}}$ solved using fixed-point Richardson iterations (Eq. (3)) with step size $\tau$, under the conditions in Eq. (11),

11 $$ 0 < \tau < \frac{2}{{\left| {{\mathbf{A}}^{T} {\mathbf{A}}} \right|_{2} }} \; {\text{and}} \; \eta < \frac{{\tau \left| {{\mathbf{A}}^{T} {\mathbf{A}}} \right|_{2} }}{{\kappa - \tau \left| {{\mathbf{A}}^{T} {\mathbf{A}}} \right|_{2} }}. $$

the asymptotic error, $\theta$, between the estimation, ${\mathbf{x}}_{k}$, and the solution, ${\mathbf{x}}^{*}$, is bounded by:

12 $$ \theta = \mathop {\lim }\limits_{k \to \infty } \sup \frac{{\left| {{\mathbf{x}}^{*} - {\mathbf{x}}_{k} } \right|_{2} }}{{\left| {{\mathbf{x}}^{*} } \right|_{2} }} \le \eta \left( {\frac{\kappa }{{\tau \left| {{\mathbf{A}}^{T} {\mathbf{A}}} \right|_{2} }} - 1} \right), $$

where $\kappa$ is the condition number of ${\mathbf{A}}^{T} {\mathbf{A}}$, and $\eta$ is the normalized *ℓ*^2^ error of the two matrix–vector multiplications in each iteration.


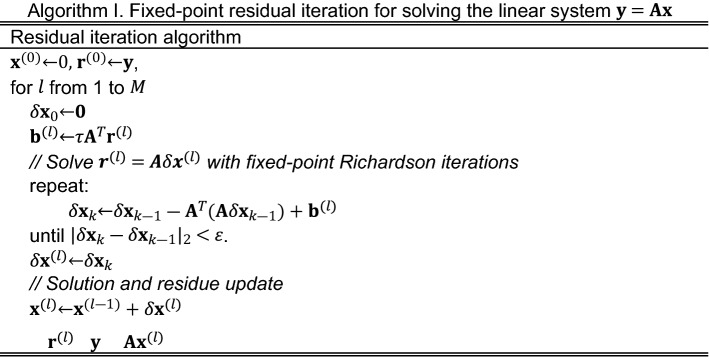


Theorem 1 is proved in Section S2 of the supplementary materials by examining the *ℓ*^2^ norm of the error of intermediate estimations, $\left| {{\mathbf{x}}^{*} - {\mathbf{x}}_{k} } \right|_{2}$, as $k \to \infty$. The proof shows a linear convergence with rate $\left| {{\mathbf{I}} - \tau {\mathbf{A}}^{T} {\mathbf{A}}/\kappa } \right|_{2}$, which is independent on the error of the fixed-point matrix–vector product, $\eta$. Because a typical problem involves a value of $\kappa$ on the order of $10^{2} \sim 10^{3}$, using the choice of step size $\tau$ in Eq. (10), $\tau \left| {{\mathbf{A}}^{T} {\mathbf{A}}} \right|_{2}$ = $2 - \chi \ll \kappa$, we can approximate

13 $$ \left| {{\mathbf{I}} - \tau {\mathbf{A}}^{T} {\mathbf{A}}/\kappa } \right|_{2}^{k} \approx \exp \left( { - \gamma k} \right), $$

where the convergence rate $\gamma = \left( {2 - \chi } \right)/\kappa$.

### Fixed-point residual iteration

The asymptotic error $\theta$ in Theorem 1 is independent of the solution ${\mathbf{x}}^{*}$, which implies that the fixed-point iterative solver can stagnate at an estimation, ${\mathbf{x}}_{\infty }$, closer to the solution ${\mathbf{x}}^{*}$ in terms of *ℓ*^2^ distance, if ${\mathbf{x}}^{*}$ has a lower *ℓ*^2^ norm. Hence, constructing a sequence of fixed-point iterative solvers in which the solutions have decreasing *ℓ*^2^ norm becomes the natural choice to approach the unbiased solution of the inverse problem.

The proposed residual iteration algorithm described in Algorithm 1 leverages the adjustable exponent of the fixed-point format. The algorithm contains $M$ sets of fixed-point Richardson iterations as the inner loops. The acceptance criterion of the inner loop solution is:

14 $$ \left| {\delta {\mathbf{x}}_{k} - \delta {\mathbf{x}}_{k - 1} } \right|_{2} < \varepsilon , $$

where $\varepsilon$ is a small number of the order $\zeta_{v}$. Let ${\mathbf{x}}^{\left( M \right)} = \mathop \sum \limits_{l = 1}^{M} \delta {\mathbf{x}}^{{\left( {\varvec{l}} \right)}}$ denote the accumulated solutions from $M$ fixed-point Richardson iterative solvers. The residual iteration algorithm aims to reduce the norm of ${\mathbf{r}}^{\left( l \right)} : = {\mathbf{y}} - {\mathbf{Ax}}^{\left( l \right)}$ at each outer loop $l$ by recursively solving the linear system ${\mathbf{r}}^{\left( l \right)} = {\mathbf{A}}\delta {\mathbf{x}}^{\left( l \right)}$ with the updated exponents in the next inner loop of the fixed-point Richardson iterations.

It is worth noting that similar precision refinement methods ^28,29^ have been employed in iterative solvers using mixed full- and half-precision floating-point data formats. Yet, the floating-point precision refinement typically requires far fewer residual loops, $M$, than Algorithm 1. In addition, floating-point format allows a different exponent for every element in the matrix–vector product. As a result, the error analysis in floating-point precision refinement ^35^ does not apply to fixed-point residual iterations. Here we present the upper bound of the normalized error of the solution ${\mathbf{x}}^{\left( M \right)}$ after $M$ fixed-point residual iterations in Theorem 2.

***Theorem 2***: For a full-rank linear system ${\mathbf{Ax}} = {\mathbf{y}}$ with its solution ${\mathbf{x}}^{*}$ obtained from the residual iteration algorithm (Algorithm 1), the asymptotic error of the estimation after $M$ residue updates, ${\mathbf{x}}^{\left( M \right)}$, is bounded by:

15 $$ \theta^{\left( M \right)} \le \theta^{M} , $$

where $\theta$ is the asymptotic error of the solution obtained from fixed-point Richardson solver (Eq. (12)).

Theorem 2 is established based on the decreasing *ℓ*^2^ norm of the solution ${\mathbf{x}}^{*\left( l \right)}$ in each fixed-point Richardson iteration. The upper bound of the solution error after $M$ residue updates can be derived recursively from Eq. (12) in Theorem 1. Detailed proof is presented in Section S3 of the supplementary materials.

For Eq. (15) to be valid, the exponents of ${\mathbf{b}}^{\left( l \right)}$ and ${\mathbf{A}}^{T} {\mathbf{A}}\delta {\mathbf{x}}_{k}$ should be updated after every iteration of the inner loop. In practice, due to the computational cost of distribution-based estimation of the infinity norm (Eq. (19)), the exponents are adjusted after the completion of each inner loop. As a result, the errors may not always satisfy the bound predicted by Eq. (15).

## Fixed-point iterative solver experiments

### Matrix inversion

**Figure 1:**
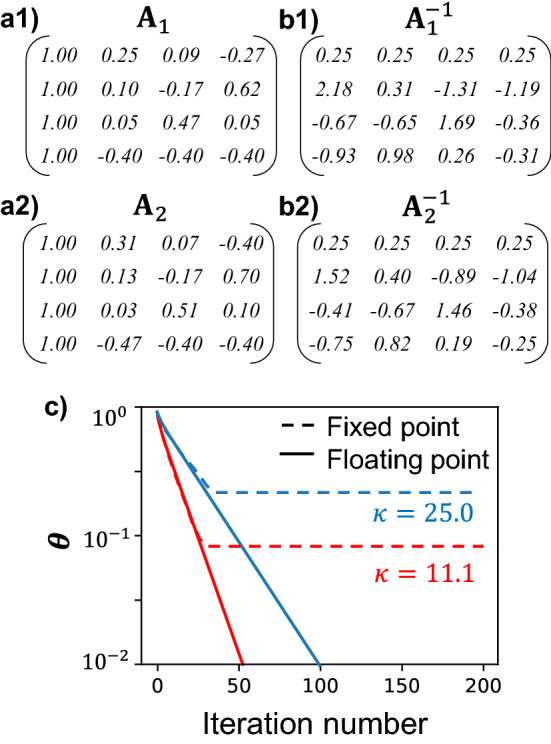
Two 4 $\times$ 4 matrices (**a**) and their inverses (**b**) in fixed-point iterative matrix inversion solver. ${\mathbf{A}}_{1}$ has a $\kappa$ of 25, and ${\mathbf{A}}_{2}$ has a $\kappa$ of 11.1. All matrices are stored in floating-point format but displayed with two decimal points. (**c**) Normalized error of the iterative solutions ${\theta }_{k}$ vs. the iteration number $k$ for the inversion of ${\mathbf{A}}_{1}$ ($\kappa$=25.0) and ${\mathbf{A}}_{2}$ ($\kappa$=11.1) using 8-bit fixed-point Richardson iterations.

We first verify the convergence rate and the residue error in theorem 1 with a fixed-point matrix inversion solver. The two matrices to be inverted, ${\mathbf{A}}_{1}$ and ${\mathbf{A}}_{2}$, shown in Fig. 1a, are constructed from the 4 $\times$ 4 discrete cosine transfer matrix by applying different linear scaling factors to its eigenvalues. The condition numbers $\kappa$ of ${\mathbf{A}}_{1}$ and ${\mathbf{A}}_{2}$ are 25.0 and 11.1, respectively. The analytical inversions ${\mathbf{A}}_{1}^{ - 1}$ and ${\mathbf{A}}_{2}^{ - 1}$, shown in Fig. 1b, are calculated with floating-point LU decomposition.

The solver uses signed 8-bit fixed-point format. The step sizes, $\tau$, are calculated from Eq. (10) with a safety margin, $\chi$=0.2, for the inversions of ${\mathbf{A}}_{1}$ and ${\mathbf{A}}_{2}$. To estimate the normalized *ℓ*^2^ error $\eta$ of the matrix–vector product, ${\mathbf{A}}^{T}\mathbf{A}{\mathbf{x}}_{k}$, the arrays ${\mathbf{A}}^{T}\mathbf{A}$ and $\mathbf{b}$ in Eq. (3) are pre-calculated and converted into fixed-point. We enumerate 200 normally distributed $\widetilde{\mathbf{x}}$ with mean $\mathbf{b}$ and standard deviation ${\left|\mathbf{b}-{\mathbf{x}}^{*}\right|}_{2}/2$, where ${\mathbf{x}}^{*}$ is the solution. The enumerated $\widetilde{\mathbf{x}}$ are converted to fixed-point format and multiplied with the fixed-point matrix ${\widetilde{\mathbf{A}}}^{T}\widetilde{\mathbf{A}}$. We then obtain $\eta$=0.019 from the average normalized root-mean-square error (RMSE) of the multiplication results.

Figure 1c plots the normalized errors, ${\theta }_{k}$, in log-scale compared with the analytical inverses, ${\mathbf{A}}_{1}^{-1}$ and ${\mathbf{A}}_{2}^{-1}$, as the iterations progress. The asymptotic errors, $\theta$, of the 8-bit iterations are 0.21 and 0.083 for the inversions of ${\mathbf{A}}_{1}$ and ${\mathbf{A}}_{2}$, respectively, both of which are consistent with the error bounds given by Eq. (12). The convergence rate, $\gamma$, is obtained from the exponential fitting of Fig. 1c with a 95% confidence level. For the inversions of ${\mathbf{A}}_{1}$ and ${\mathbf{A}}_{2}$, $\gamma$ is 0.040 ± 0.001 and 0.085 ± 0.004 respectively, which are inversely proportional to $\kappa$, as predicted by Eq. (13). The asymptotic errors and convergence rates are summarized in Table 1.

**Table 1:** Normalized errors and the convergence rate of the 8-bit Richardson iterations used to obtain the inverse of ${\mathbf{A}}_{1}$ and ${\mathbf{A}}_{2}$. A 95% confidence bound is used in exponential fitting.

Matrix to invert	$\kappa$	$\theta$	Upper bound of $\theta$	Convergence rate $\gamma$
${\mathbf{A}}_{1}$	25.0	0.21	0.24	0.040 ± 0.001
${\mathbf{A}}_{2}$	11.1	0.083	0.098	0.085 ± 0.004

**Figure 2:**
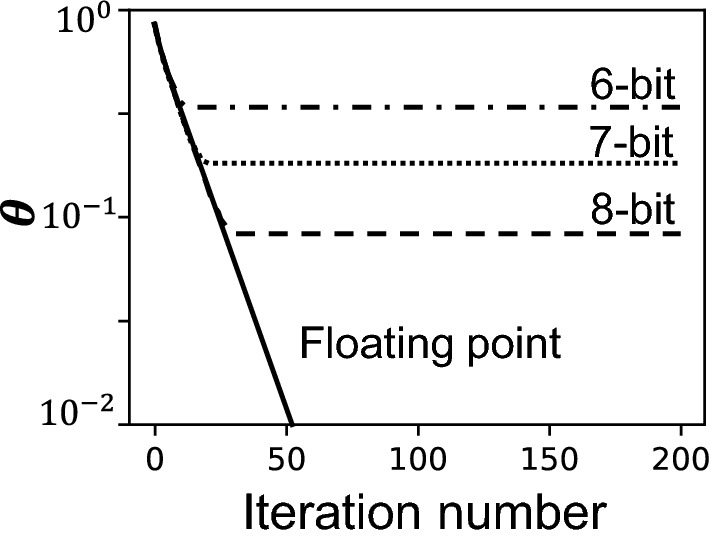
Normalized errors of the iterative solutions ${\theta }_{k}$ vs. the iteration number $k$ for the inversion of ${\mathbf{A}}_{2}$ ($\kappa$=11.1) using 8-bit, 7-bit, and 6-bit fixed-point iterative solvers. The error of the floating-point iterative solver is plotted for reference.

For the same condition number, Fig. 2 compares the fixed-point Richardson iterations with the 8-bit, 7-bit, 6-bit, and floating-point precisions. The convergence rates are summarized in Table 2, which confirms the implication of Eq. (13) that the fixed-point iterative solver maintains the same convergence rate, $\gamma$, as the floating-point counterpart. The independence of convergence rate on the precision suggests that fixed-point iterative solvers do not compromise the convergence speed. The asymptotic errors of the 8-bit, 7-bit, and 6-bit solutions are 0.083, 0.18, and 0.33, respectively, which are all below the upper bound established in Eq. (12).

**Table 2:** Asymptotic errors and convergence rates of the 8-bit, 7-bit, 6-bit, and floating-point iterations. A 95% confidence bound is used in exponential fitting.

Precision	$\eta$	$\theta$	Upper bound of $\theta$	Convergence rate $\gamma$
8-bit	0.019	0.083	0.098	0.085 ± 0.002
7-bit	0.036	0.18	0.19	0.085 ± 0.004
6-bit	0.072	0.33	0.37	0.088 ± 0.009
Floating-point	N.A	N.A	N.A	0.083 ± 0.003

### Image deconvolution

Deconvolution is a classic linear inverse problem that has broad applications in signal/image filtering and processing. For a linear, shift-invariant system, the convolution output, ${\mathbf{y}}$ (i.e., filtered signal, or blurred image) is the discrete convolution between the input signal or image ${\mathbf{x}}$ and the kernel ${\mathbf{K}}$, i.e., ${\mathbf{y}} = {\mathbf{K}}*{\mathbf{x}}$, which is defined elementwise as:

16 $$ y_{i,j} = \mathop \sum \limits_{p,q = 0}^{M,N} K_{p,q} x_{i - p,j - q} , $$

where $M$ and $N$ are the sizes of the kernel along row and column directions, respectively. Here, we have assumed a two-dimensional convolution, as in the case of image processing. This example demonstrates the iterative Richardson-Lucy deconvolution, which recovers images from blurry measurements given the convolution kernel.

The discrete convolution can be formulated as the product between the vectorized input, ${\mathbf{x}}$, and a Toeplitz matrix, ${\mathbf{A}}$, constructed from the kernel ^36^, producing the output ${\mathbf{y}} = {\mathbf{Ax}}$ in the vectorized form. To estimate the condition number, we rewrite Eq. (16) as Eq. (17) following the convolution theorem,

17 $$ {\mathbf{y}} = {\mathbf{\mathcal{F}}}^{\dag } {\text{diag}}\left( {{\tilde{\mathbf{K}}}} \right){\mathbf{\mathcal{F}}}{\mathbf{x}}, $$

where ${\mathbf{\mathcal{F}}}$ and ${\mathbf{\mathcal{F}}}^{\dag }$ represent the forward and inverse two-dimensional Fourier transforms, both of which are unitary operators, $\dag$ denotes the conjugate transpose of a complex matrix, and ${\text{diag}}\left( {{\tilde{\mathbf{K}}}} \right)$ is the diagonal matrix constructed from the Fourier transform of the kernel ${\tilde{\mathbf{K}}}$. The condition number, $\kappa$, of ${\mathbf{A}}^{T} {\mathbf{A}}$ can be obtained from the maximum and minimum elements in ${\text{diag}}\left( {{\tilde{\mathbf{K}}}} \right)^{\dag } {\text{diag}}\left( {{\tilde{\mathbf{K}}}} \right)$.

**Figure 3:**
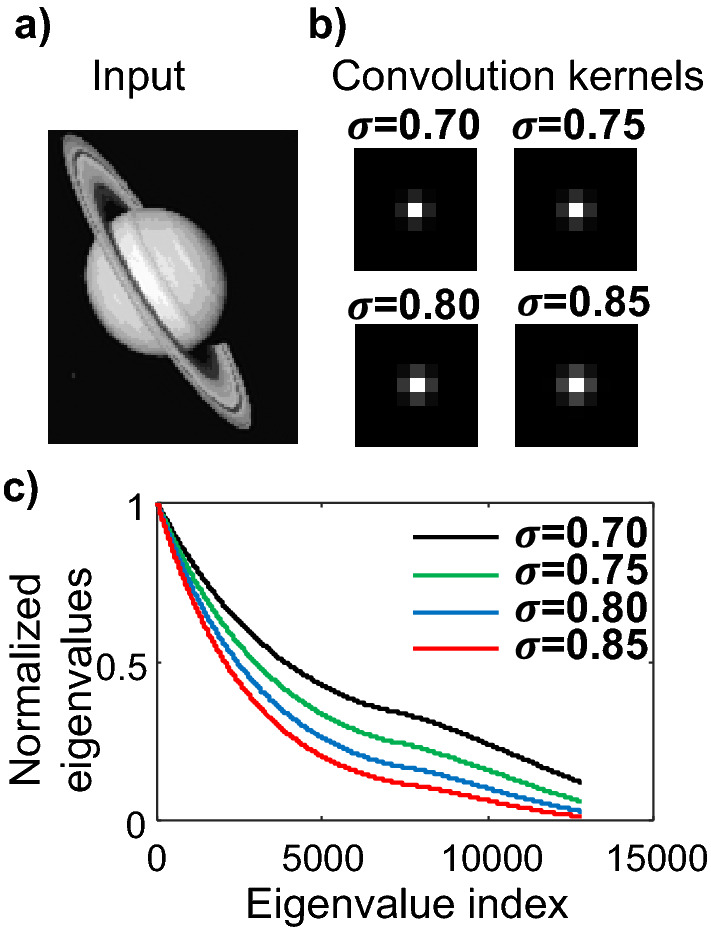
Fixed-point iterative solver for a discrete Richardson-Lucy deconvolution problem. (**a**) The 128 $\times$ 102 signed 4-bit input image “Saturn”. (**b**) Four convolution kernels with $\sigma$=0.70, 0.75, 0.80, and 0.85, respectively. (**c**) Eigenvalue spectra of the Toeplitz matrices calculated from the Fourier transforms of the convolution kernels. The condition numbers, $\kappa$, of ${\mathbf{A}}^{T}\mathbf{A}$ are 8.38, 16.59, 35.07, and 78.50, respectively.

Figure 3 shows an example based on Richardson-Lucy deconvolution. The input, ${\mathbf{x}}^{*}$, is a test image downsampled from the MATLAB image “Saturn”, shown in Fig. 3 (a). The image contains128 × 102 pixels in signed 4-bit fixed-point format with range (− 1,1). The convolution kernel, ${\mathbf{K}}$, follows the Gaussian profile,

18 $$ K_{p,q} = K_{0} \exp \left( { - \frac{{\left( {p - \frac{M}{2}} \right)^{2} + \left( {q - \frac{N}{2}} \right)^{2} }}{{2\sigma^{2} }}} \right), $$

where the parameter $\sigma$ determines the width of the kernel, and $K_{0}$ is a normalization constant that ensures that $\mathop \sum \limits_{p,q} \left| {K_{p,q} } \right|^{2} = 1$. Four example kernels with $\sigma$ = 0.70, 0.75, 0.80, and 0.85, are plotted in Fig. 3b. Figure 3c shows the normalized eigenvalue spectra of the four Toeplitz matrices, calculated from the Fourier transform of the kernels. The condition numbers, $\kappa$, of the four kernels are 8.38, 16.59, 35.07, and 78.50, respectively. If $\sigma$ is increased beyond 0.90, $\kappa$ would violate the criteria in Eq. (11) and lead to a non-converging deconvolution result. Similar phenomenon has been reported in hybrid analog–digital solvers ^37^.

**Figure 4:**
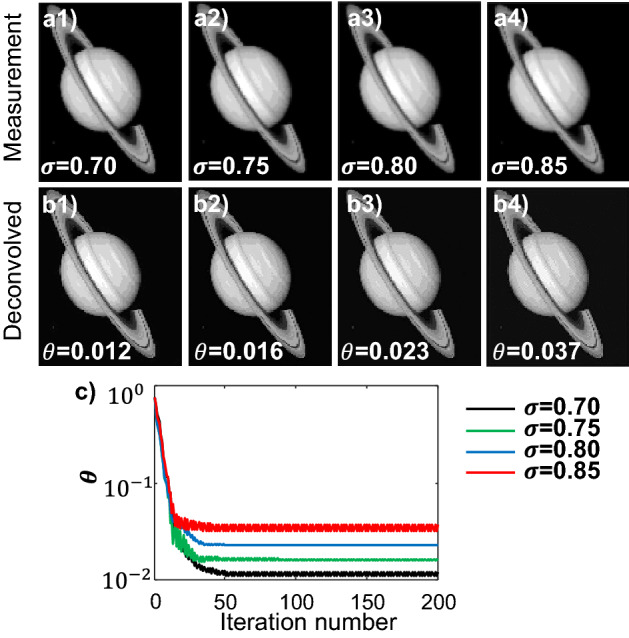
8-bit fixed-point Richardson solver of a discrete Richardson-Lucy deconvolution problem. (**a**) Convolution measurements from the four kernels with $\sigma$ of (a1) 0.70, (a2) 0.75, (a3) 0.80, and (a4) 0.85. (b1-b4) Deconvolved images using 8-bit fixed-point iterations from (a1-a4). (**c**) Normalized error vs. iteration steps for the reconstructions in (b1-b4).

The measurements, shown in Fig. 4a, are calculated from convolving the input image, ${\mathbf{x}}^{*}$, with the four kernels in Fig. 3b, and then quantized to 8-bit to simulate the digitization error of an ordinary CCD or CMOS camera. The deconvolution problems are solved using 8-bit fixed-point iterations, with $\eta$ estimated to be 0.014. Step sizes with the safety margin $\chi$ = 0.2 are used for all four iterative deconvolutions. Figure 4b plots the deconvolved images after 200 iterations. The normalized errors as functions of iteration steps are plotted in Fig. 4c. The asymptotic errors, $\theta$, are summarized in Table 3, and are all below the error bound predicted by Eq. (12).

**Table 3:** Normalized errors of Richardson-Lucy deconvolution results.

$\sigma$	$\kappa$	$\theta$	Upper bound of $\theta$
0.70	8.38	0.012	0.056
0.75	16.59	0.016	0.12
0.80	35.07	0.023	0.25
0.85	78.50	0.037	0.53

### Tomographic reconstruction with residual iteration

**Figure 5:**
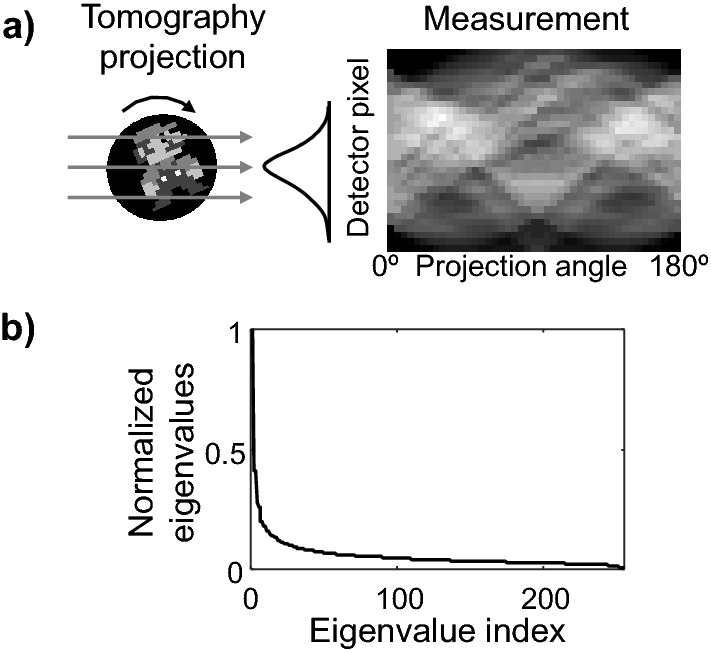
Fixed-point iterative solver for a tomographic reconstruction problem. (**a**) Tomographic projection and measurement of a 16 $\times$ 16 signed 3-bit “Super Mario” pixel art. (**b**) Eigenvalue spectrum of the tomographic projection model. The condition number $\kappa$ of ${\mathbf{A}}^{T}\mathbf{A}$ is 101.80.

Theorem 2 indicates that the residual iteration can overcome the stagnation of the fixed-point Richardson solver and reach a solution beyond the fixed-point limit. To verify the performance of the residual iterations, we examine a tomographic reconstruction of a 16 × 16 “Super Mario” pixel art from its pencil-beam projections, shown in Fig. 5a. The pixels of the input image, ${\mathbf{x}}^{*}$, are stored in the signed 3-bit fixed-point format with range (− 1,1), giving a quantization interval of 0.25 between two adjacent intensity levels. The measurements consist of 60 projections between 0 and 180º at 4º intervals, each containing 31 uniformly spaced beams. The system matrix, ${\mathbf{A}}$, of the tomographic model is constructed from the distance-driven method ^38^. Figure 5b plots the eigenvalue spectrum of the tomographic model. The condition number, $\kappa$, of ${\mathbf{A}}^{T} {\mathbf{A}}$ is 101.80. Assuming the maximum step size, $\tau$, is used in the Richardson iterations, the computation error $\eta$ must be less than 0.020 to ensure convergence, according to Eq. (11). For the size and sparsity of this system matrix, we find that a minimum of $L$ = 8 bits (with an estimated $\eta$ = 0.015) are required for fixed-point iterations to converge.

Fixed-point Richardson and residual iterations with 8-bit, 9-bit, and 10-bit precisions are performed to reconstruct the “Super Mario” pixel art from the tomographic projections. The step sizes, $\tau$, with safety margin $\chi$ = 0.3 are used in all the solvers. The system matrix ${\mathbf{A}}$ is quantized to the solver precision with expo = 0. The measurement, ${\mathbf{y}}$, is serialized to a vector, multiplied by $\tau {\mathbf{A}}^{T}$, and quantized to the solver precision with expo = 0 to pre-calculate the fixed-point vector ${\mathbf{b}}$ for the iterations. The fixed-point Richardson iteration sets the exponent of the multiplication between ${\mathbf{A}}^{T} {\mathbf{A}}$ and ${\mathbf{x}}_{k}$ to 2. For residual iterations, the exponents of matrix–vector multiplications are decremented by 1 every 80 steps from 2 to − 2.

**Figure 6:**
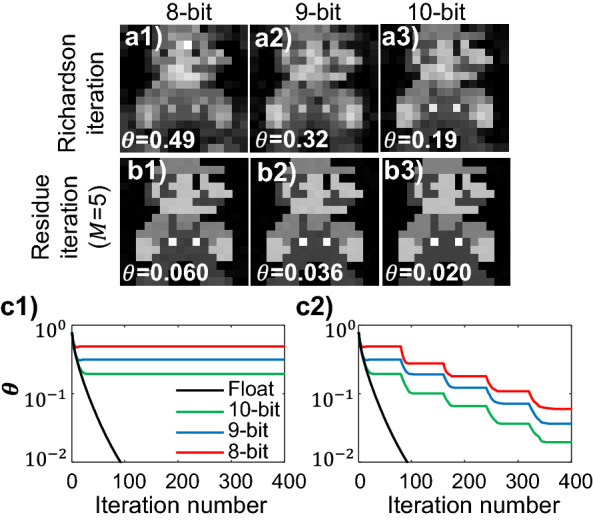
Richardson (**a**) and residual (**b**) iterations for reconstructing the “Super Mario” pixel art from tomographic measurements. (**a**) Reconstructions from 8-bit (a1), 9-bit (a2), and 10-bit (a3) Richardson iterations. (**b**) Reconstructions from 8-bit (b1), 9-bit (b2), and 10-bit (b3) residual iterations after $M$=5 residue update steps. (**c**) Normalized error vs. iteration steps for 8-bit (red curves), 9-bit (blue curves), and 10-bit (green curves) Richardson (c1) and residual (c2) iterations. The floating-point (black curve) reconstruction is plotted on both (c1) and (c2) for reference.

Figure 6 plots the reconstructions from Richardson and residual iterations with 8-bit, 9-bit, and 10-bit precisions. Table 4 summarizes the normalized errors of the fixed-point Richardson reconstructions, all of which are below the error bound given by Eq. (12). The normalized error, $\theta$, is plotted as a function of the Richardson iteration number in Fig. 6c1. Note that the same convergence rate is present regardless of the precision of the solver. The normalized errors of the residual iterations are shown in Fig. 6c2. After $M$ = 5 residue updates, the 8-bit, 9-bit, and 10-bit solvers all reach normalized errors below 0.1. At this level of $\theta$, the pixelwise differences between ${\mathbf{x}}^{\left( 5 \right)}$ and ${\mathbf{x}}^{*}$ are below the quantization interval, 0.25, of the true image ${\mathbf{x}}^{*}$. Therefore, the reconstructions in Fig. 6b1–b3 would exhibit no visual differences from the true image if displayed in 3-bit grayscale colormap.

**Table 4:** Normalized errors of 8-bit, 9-bit, and 10-bit Richardson iterations.

Precision	$\eta$	$\theta$	Upper bound of $\theta$
8-bit	0.015	0.49	0.88
9-bit	0.0056	0.32	0.33
10-bit	0.0028	0.19	0.16

**Figure 7:**
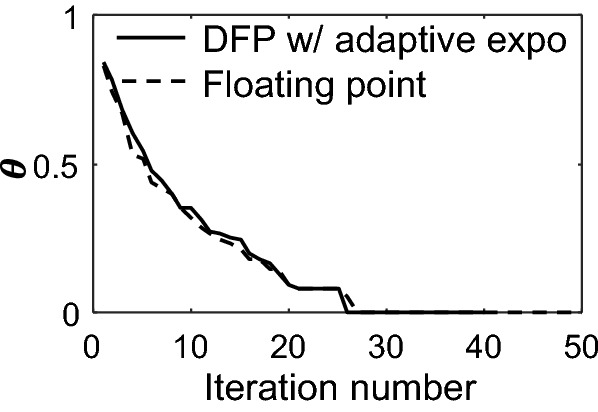
Normalized error vs. iteration number for the floating-point solver (dashed curve) and fixed-point iterative solver (solid curve), with adaptive exponent adjustments made every 5 steps.

Faster convergence is possible via adaptive adjustments of the exponents of ${\mathbf{b}}^{\left( l \right)}$ and ${\mathbf{A}}^{T} {\mathbf{A}}\delta {\mathbf{x}}_{k}$. Figure 7 shows the error of the 8-bit residual iteration executed on the hardware prototype solver (Sec. "Hardware prototype for a fixed-point iterative solver"). The exponent is updated every 5 steps based on the distribution of the elements in a fixed-point array ${\mathbf{x}}$. The exponents are calculated using:

19 $$  {\text{expo}} = \left\lceil {\log _{2} \left( {\left| {\mu _{{\mathbf{x}}} } \right| + 3\sigma _{{\mathbf{x}}} } \right)} \right\rceil ,  $$

where $\mu_{{\mathbf{x}}}$ and $\sigma_{{\mathbf{x}}}$ denote the mean and standard deviation, respectively, of all the elements in ${\mathbf{x}}$. The adaptive residual iteration achieves the same convergence speed as the floating-point solver, with both methods achieving errors below the quantization interval of the true solution after 28 iterations.

## Hardware prototype for a fixed-point iterative solver

**Figure 8:**
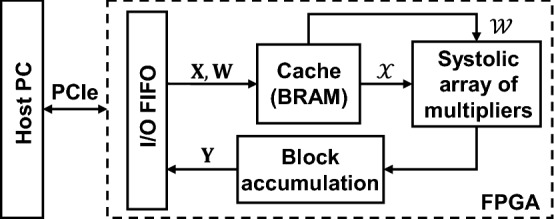
Block diagram of the fixed-point iterative solver prototype on FPGA depicting the communications and among systolic multiplier array, cache, accumulator, and other resources. FIFO: first-in-first-out; BRAM: block random-access memory.

We have built a hardware prototype to perform the fixed-point Richardson iteration and the adjustment of exponent. The core component of the solver, i.e., the fixed-point matrix multiplication, is implemented on an FPGA development board (Xilinx ZC706) and communicates with the host PC via PCIe bus. Figure 8 illustrates the block diagram of the logic design within the FPGA. The systolic multiplier array performs 512 MAC operations in a single clock cycle. The inputs of the multiplier array consist of a 16 × 16 ${\mathcal{W}}$ block and a 16 × 2 ${\mathcal{X}}$ block. The precisions of both input blocks are signed 8-bit. The multiplication outputs are cached in signed 32-bit and wired to a 16 × 2 array of accumulators. For the matrix–vector multiplication involving a matrix ${\mathbf{W}}$ larger than 16 × 16, the matrix ${\mathbf{W}}$ is zero-padded to an integer number of 16 × 16 ${\mathcal{W}}$ blocks, and the batch vector ${\mathbf{X}}$ is zero-padded to an integer number of 16 × 2 ${\mathcal{X}}$ blocks, along the row dimension only. All the decomposed ${\mathcal{W}}$ and ${\mathcal{X}}$ blocks are cached in on-chip block random access memory (BRAM). The multiplier array reads the ${\mathcal{W}}$ blocks along the rows and the ${\mathcal{X}}$ blocks along the columns. The blocks of 16 × 2 results are summed in the accumulators. After completion of one row of ${\mathcal{W}}$ blocks, the multiplier array reads the ${\mathcal{W}}$ blocks in the next row and cycles through all the blocks of ${\mathcal{X}}$ again.

The host PC converts ${\mathbf{W}}$ and ${\mathbf{X}}$ into fixed-point, and then decomposes the mantissas into 16 × 16 ${\mathcal{W}}$ blocks and 16 × 2 ${\mathcal{X}}$ blocks. The decomposed ${\mathcal{W}}$ and ${\mathcal{X}}$ blocks are streamed to the FPGA through PCIe. Once all the blocks are multiplied and accumulated in FPGA, the results ${\mathbf{Y}}$ are streamed back to host PC in 32-bit integer format. The host PC masks the results back to 8-bit, with the most significant bit (MSB) and least significant bit (LSB) selected by the exponents. The host PC updates the exponents according to Eq. (19) every five iterations.

For the FPGA design in Fig. 8 implemented on the ZC706 evaluation board, the Xilinx synthesis and implementation tools estimate a power consumption of 2.047W, of which 1.253W is consumed by the transceivers for PCIe communication with the host PC, and the remaining 0.794W is consumed by the logics, including the clocks, systolic multiplier array, BRAM cache, and control units that generate the addresses of ${\mathcal{W}}$ and ${\mathcal{X}}$ blocks. The clock rate is 250 MHz. Considering the total number of 512 MACs within a single clock cycle, the energy consumption by the fixed-point computation is 6.2pJ/MAC, which is on the same order as that of datacenter TPUs ^39^.

We have tested the speed of the iterative solver implemented on our hardware prototype and CPU (Intel Core i3-4130, dual-core, 3.40 GHz clock rate). The total CPU time of the fixed-point Richardson iterations, excluding exponent adjustment, is measured, and then divided by the total number of iterations in all residual updates to obtain the time per iteration, which is indicative of the calculation speed. The fixed-point solver on CPU takes 1.7 ± 0.2 ms per iteration, while that on FPGA prototype takes 0.76 ± 0.05 ms per iteration, representing more than two times of improvement in speed. Errors represent the variance of the calculation time within five repeated tests.

## Conclusion

We have demonstrated a fixed-point iterative solver that computes high-precision solutions for linear inverse problems beyond the precision limit of the hardware. We have described the convergence and error of the fixed-point iterations in two theorems. Theorem 1 shows that the convergence rate is independent of the precision of the solver, which implies that fixed-point iteration does not compromise the convergence speed of the solver. Theorem 2 resolves the stagnation of a fixed-point solver with residual iterations, which correct the error and refine the solution to a higher precision level. We have presented three examples of linear inverse problems, namely, matrix inversion, Richardson-Lucy deconvolution, and tomographic reconstruction, to verify both theorems.

The combination of residual iteration with adaptive exponent adjustment achieves the same rate constant and error as a floating-point Richardson solver. We have prototyped the fixed-point solver with a fixed-point systolic multiplier array on the FPGA and fixed-point arithmetic libraries on host PC. The prototype solver achieves more than twice the calculation speed of a CPU and takes the same number of iterations to reach the solution as its floating-point counterpart. We envision that an array of low-precision, analog matrix processing cores can supersede the current FPGA systolic array in solving numerous large-scale, high-performance computing applications, including but not limited to convex optimizations, solving differential equations, and training of artificial neural networks. Certain nonlinear problems ^40,41^ could also be incorporated into our iterative solver framework in conjunction with digital co-processing to calculate the local gradient on host PC.

## Supplementary Information

Supplementary Information.

## Data Availability

The data and software that support the findings of this study are available from the corresponding author upon reasonable request.
