# Supplementary material for: Fixed-point iterative linear inverse solver with extended precision
Source: Sci Rep. 2023 Mar 30;13:5198. doi: 10.1038/s41598-023-32338-5 (PMC10063671; doi:10.1038/s41598-023-32338-5)
Supplement: Supplementary file 1 — Supplementary Information. [file 41598_2023_32338_MOESM1_ESM.docx]

Supplementary materials for “Fixed-point iterative linear inverse solver with extended precision”

S1. Upper bound of matrix-vector multiplication error

We derive the upper bound of the normalized error, $\eta$ for fixed-point matrix-vector multiplication. Based on Eq. (5), for an $N$-dimensional vector, $\mathbf{x}$, the infinity norm falls in the range:

|  | $2^{\mathrm{expo}-1}\leq\left\vert\mathbf{x} \right\vert_{\infty}\leq2^{\mathrm{expo}}.$ | (S1) |
| --- | --- | --- |

The infinity norm of fixed-point rounding error, $\left| \mathbf{x-} \tilde{\mathbf{x}} \right|_{\infty}$, is determined by the bit width:

|  | $0\leq\left\vert\mathbf{x-} \tilde{\mathbf{x}} \right\vert_{\infty}\leq2^{expo-\left( L-1 \right)}.$ | (S2) |
| --- | --- | --- |

The equivalence of norms establishes the inequality between infinity norm and *ℓ*^2^ norm, $\left| \mathbf{x} \right|_{\infty}\leq\left| \mathbf{x} \right|_{2}\leq\sqrt{N}\left| \mathbf{x} \right|_{\infty}$. This gives the normalized *ℓ*^2^ error, $\zeta_{v}=\left| \mathbf{x-} \tilde{\mathbf{x}} \right|_{2}/\left| \mathbf{x} \right|_{2}$, of a fixed-point vector $\tilde{\mathbf{x}}$,

|  | $0\leq\zeta_{v}\leq2^{2-L}\sqrt{N}.$ | (S3) |
| --- | --- | --- |

For an $N\times N$ matrix $\mathbf{M}$, its operator norm is used in error analysis. Using the same fixed-point conversions (Eq. (4) – (6) in the main text), the infinity norm of a fixed-point matrix $\left| \mathbf{M} \right|_{\infty}$ falls in the range:

|  | $2^{\mathrm{expo}-1}N\leq\left\vert\mathbf{M} \right\vert_{\infty}\leq2^{\mathrm{expo}}N.$ | (S4) |
| --- | --- | --- |

The infinity norm of the rounding error, $\left| \mathbf{M-} \tilde{\mathbf{M}} \right|_{\infty}$, is determined by both bit width and size, and falls in the range:

|  | $0\leq\left\vert\mathbf{M-} \tilde{\mathbf{M}} \right\vert_{\infty}\leq2^{expo-\left( L-1 \right)}N.$ | (S5) |
| --- | --- | --- |

The equivalence of norms for matrix yields$\left| \mathbf{M} \right|_{\infty}/\sqrt{N}\leq\left| \mathbf{M} \right|_{2}\leq\sqrt{N}\left| \mathbf{M} \right|_{\infty}$ [1], providing the boundaries of the normalized *ℓ*^2^ error, $\zeta_{m}=\left| \mathbf{M-} \tilde{\mathbf{M}} \right|_{2}/\left| \mathbf{M} \right|_{2}$, of a fixed-point matrix $\tilde{\mathbf{M}}$:

|  | $0\leq\zeta_{m}\leq2^{2-L}N.$ | (S6) |
| --- | --- | --- |

We introduce the rounding errors of the fixed-point matrix $\mathbf{E}=\mathbf{M}-\tilde{\mathbf{M}}$ and the vector $\mathbf{e}=\mathbf{x}-\tilde{\mathbf{x}}$. Using Eq. (S3) and Eq. (S6), the *ℓ*^2^ norm of the error $\delta\mathbf{y}=\tilde{\mathbf{M}}\tilde{\mathbf{x}}-\mathbf{Mx}$ is bounded by,

| $\left\vert\boldsymbol{\delta y} \right\vert_{2}\leq\left\vert\tilde{\mathbf{M}}\mathbf{e} \right\vert_{2}+\left\vert\mathbf{E}\tilde{\mathbf{x}} \right\vert_{2}+\left\vert\mathbf{Ee} \right\vert_{2}\leq\left\vert\tilde{\mathbf{M}} \right\vert_{2}\left\vert\mathbf{e} \right\vert_{2}+\left\vert\mathbf{E} \right\vert_{2}\left\vert\tilde{\mathbf{x}} \right\vert_{2}+\left\vert\mathbf{E} \right\vert_{2}\left\vert\mathbf{e} \right\vert_{2}=\zeta_{v}\left\vert\tilde{\mathbf{M}} \right\vert_{2}\left\vert\mathbf{x} \right\vert_{2}+\zeta_{m}\left\vert\mathbf{M} \right\vert_{2}\left\vert\tilde{\mathbf{x}} \right\vert_{2}+\zeta_{v}\zeta_{m}\left\vert\mathbf{M} \right\vert_{2}\left\vert\mathbf{x} \right\vert_{2}\leq\zeta_{v}{\left( 1+\zeta_{m} \right)\left\vert\mathbf{M} \right\vert}_{2}\left\vert\mathbf{x} \right\vert_{2}+\zeta_{m}{\left( 1+\zeta_{v} \right)\left\vert\mathbf{M} \right\vert}_{2}\left\vert\mathbf{x} \right\vert_{2}+\zeta_{v}\zeta_{m}\left\vert\mathbf{M} \right\vert_{2}\left\vert\mathbf{x} \right\vert_{2}=\left( \zeta_{v}+\zeta_{m}+3\zeta_{v}\zeta_{m} \right)\left\vert\mathbf{M} \right\vert_{2}\left\vert\mathbf{x} \right\vert_{2}.$ | (S7) |
| --- | --- |

Let us define the normalized error of the matrix-vector product as,

|  | $\eta:=\frac{\left\vert\boldsymbol{\delta y} \right\vert_{2}}{\left\vert\mathbf{M} \right\vert_{2}\left\vert\mathbf{x} \right\vert_{2}},$ | (S8) |
| --- | --- | --- |

the upper bound of $\eta$ is $\zeta_{v}+\zeta_{m}+3\zeta_{v}\zeta_{m}$.

S2. Proof of Theorem 1

We prove Theorem 1 by examining the *ℓ*^2^ error of the intermediate estimates $\left| \mathbf{x}^{*}-\mathbf{x}_{k} \right|_{2}$. Without loss of generality, the normalized *ℓ*^2^ error $\eta$ of two matrix-vector multiplications can be combined into one multiplication error $\boldsymbol{\delta y}$ between a matrix, $\mathbf{B:=I}-\tau\mathbf{A}^{T}\mathbf{A}$, and the vector $\mathbf{x}_{k}$. Since $\mathbf{x}^{*}\mathbf{=B}\mathbf{x}^{*}+\mathbf{b}$, the error of the solution at iteration $k$ can be obtained recurrently,

| $\mathbf{x}^{*}-\mathbf{x}_{k}=\mathbf{B}\left( \mathbf{x}^{*}-\mathbf{x}_{k-1} \right)-\boldsymbol{\delta y}=\mathbf{B}^{2}\left( \mathbf{x}^{*}-\mathbf{x}_{k-2} \right)-\left( \boldsymbol{B\delta y}+\boldsymbol{\delta y} \right)=\ldots=\mathbf{B}^{k}\left( \mathbf{x}^{*}-\mathbf{x}_{0} \right)-\sum_{p=0}^{k-1} \left( \mathbf{B} \right)^{p}\boldsymbol{\delta y}.$ | (S9) |
| --- | --- |

Assuming the initial guess, $\mathbf{x}_{0}=\mathbf{0}$, the *ℓ*^2^ norm of the error at iteration $k$ is,

| $\left\vert\mathbf{x}^{*}-\mathbf{x}_{k} \right\vert_{2}\boldsymbol{\leq}\left\vert\mathbf{B}^{k}\mathbf{x}^{*} \right\vert_{2}\mathbf{+}\left\vert\sum_{p=0}^{k-1} \left( \mathbf{B} \right)^{p}\boldsymbol{\delta y} \right\vert_{2}\boldsymbol{\leq}\left\vert\mathbf{B} \right\vert_{2}^{k}\left\vert\mathbf{x}^{*} \right\vert_{2}+\sum_{p=0}^{k-1} \left\vert\left( \mathbf{B} \right) \right\vert_{2}^{p}\left\vert\boldsymbol{\delta y} \right\vert_{2}\leq\left\vert\mathbf{B} \right\vert_{2}^{k}\left\vert\mathbf{x}^{*} \right\vert_{2}+\frac{1-\left\vert\mathbf{B} \right\vert_{2}^{k}}{1-\left\vert\mathbf{B} \right\vert_{2}}\left\vert\boldsymbol{\delta y} \right\vert_{2}.$ | (S10) |
| --- | --- |

Substituting the matrix-vector product error $\left| \boldsymbol{\delta y} \right|$ with the upper bound $\eta\left| \mathbf{B} \right|\left| \mathbf{x}^{*} \right|$ (Eq. (7) in main text), the normalized error of the solution, $\theta_{k}$, at iteration $k$ is bounded by,

| $\theta_{k}=\frac{\left\vert\mathbf{x}^{*}-\mathbf{x}_{k} \right\vert_{2}}{\left\vert\mathbf{x}^{*} \right\vert_{2}}\leq\frac{\eta\left\vert\mathbf{B} \right\vert_{2}}{1-\left\vert\mathbf{B} \right\vert_{2}}+\left( 1-\frac{\eta\left\vert\mathbf{B} \right\vert_{2}}{1-\left\vert\mathbf{B} \right\vert_{2}} \right)\left\vert\mathbf{B} \right\vert_{2}^{k}\boldsymbol{,}$ | (S11) |
| --- | --- |

where the first term is the asymptotic error, and the second term is a function of the iteration step $k$. To ensure $\theta_{k}$ decays as the iteration progresses, the convergence criteria of the Richardson iteration must be satisfied,

|  | $\left\{ \begin{aligned} -1\boldsymbol{<}\left\vert\mathbf{B} \right\vert_{2}\boldsymbol{<}1 \\ 1-\frac{\eta\left\vert\mathbf{B} \right\vert_{2}}{1-\left\vert\mathbf{B} \right\vert_{2}}>0 \end{aligned} \right.\boldsymbol{.}$ | (S12) |
| --- | --- | --- |

The largest and the smallest eigenvalues in $\mathbf{B}$ are $1-\tau\left| \mathbf{A}^{T}\mathbf{A} \right|_{2}/\kappa$ and $1-\tau\left| \mathbf{A}^{T}\mathbf{A} \right|_{2}$, respectively. Plugging $\left| \mathbf{B} \right|_{2}$ in the convergence criteria (Eq.(S12)) confirms the conditions for convergence (Eq. (11) in the main text). Using the maximum eigenvalue $\left| \mathbf{B} \right|_{2}=1-\tau\left| \mathbf{A}^{T}\mathbf{A} \right|_{2}/\kappa$ in the first term of Eq. (S11) gives the upper bound of asymptotic error $\theta$ in Theorem 1. □

S3. Proof of Theorem 2

Because the *ℓ*^2^ norm of the error is proportional to the *ℓ*^2^ norm of the solution, as indicated by Theorem 1, we prove Theorem 2 by showing that the *ℓ*^2^ norm of the solution is decaying according to $\theta^{M}$ after $M$ residue updates.

The solution to the first iteration of the outer loop ($l$=1) is $\mathbf{x}^{(1)}=\mathbf{x}^{*}$. The first outer loop produces an estimate $\delta\mathbf{x}$, with an error $\left| \mathbf{x}^{*}-\delta\mathbf{x}^{(1)} \right|_{2}$ bounded as in Theorem 1. The second iteration of the outer loop solves the linear system $\mathbf{r}^{\left( 1 \right)}=\mathbf{A}\delta\mathbf{x}$, where $\mathbf{r}^{\left( 1 \right)}=\mathbf{y}-\mathbf{A}\delta\mathbf{x}^{\left( 1 \right)}$, and its solution is $\mathbf{x}^{(2)}=\mathbf{x}^{*}-\delta\mathbf{x}^{(2)}$, whose *ℓ*^2^ norm is bounded by $\left| \mathbf{x}^{\left( 2 \right)} \right|_{2}\leq\theta\left| \mathbf{x}^{*} \right|_{2}$. Using Theorem 1, the *ℓ*^2^ norm of the error between the solution from the second set of Richardson iterations $\delta\mathbf{x}^{(2)}$ and its solution $\mathbf{x}^{(2)}$ is bounded by,

|  | $\left\vert\mathbf{x}^{\left( 2 \right)}-\delta\mathbf{x}^{\left( 2 \right)} \right\vert_{2}\leq\theta\left\vert\mathbf{x}^{\left( 2 \right)} \right\vert\leq\theta^{2}\left\vert\mathbf{x}^{*} \right\vert_{2}.$ | (S13) |
| --- | --- | --- |

Rewriting $\left| \mathbf{x}^{\left( 2 \right)}-\delta\mathbf{x}^{\left( 2 \right)} \right|_{2}$ as $\left| \mathbf{x}^{*}-\delta\mathbf{x}^{\left( 1 \right)}-\delta\mathbf{x}^{\left( 2 \right)} \right|_{2}=\left| \mathbf{x}^{*}-\mathbf{x}^{\left( 1 \right)} \right|_{2}$, the accumulated solution $\mathbf{x}^{(2)}=\delta\mathbf{x}^{\left( 1 \right)}+\delta\mathbf{x}^{(2)}$ has an error $\left| \mathbf{x}^{*}-\mathbf{x}^{\left( 1 \right)} \right|_{2}\leq\theta^{2}\left| \mathbf{x}^{*} \right|_{2}$, which is at least $\theta$ times smaller than the solution from the first iteration of the outer loop $\delta\mathbf{x}^{(1)}$. After $M$ iterations of outer loop, $\left| \mathbf{x}^{*}-\mathbf{x}^{\left( M-1 \right)} \right|_{2}\leq\theta^{M}\left| \mathbf{x}^{*} \right|_{2}$. □
